# Inequities in food access during the COVID19 pandemic: a multilevel, mixed methods pilot study

**DOI:** 10.21203/rs.3.rs-4714565/v1

**Published:** 2024-08-09

**Authors:** Megha R. Aepala, Alice Guan, Tessa Cruz, Jamaica Sowell, Brenda Mattias, Katherine Lin, Analena Hope Hassberg, Salma Shariff-Marco, Mindy C. DeRouen, Antwi Akom

**Affiliations:** Department of Epidemiology and Biostatistics | University of California, San Francisco; Department of Epidemiology and Biostatistics | University of California, San Francisco; The Social Innovation and Urban Opportunity Lab, Streetwyze | UCSF & San Francisco State University | Oakland, CA; Roots Community Health Center | Oakland, CA; The Social Innovation and Urban Opportunity Lab, Streetwyze | UCSF & San Francisco State University | Oakland, CA; Department of Epidemiology and Biostatistics | University of California, San Francisco; Department of Sociology | California State University, Los Angeles; Department of Epidemiology and Biostatistics | University of California, San Francisco; Department of Epidemiology and Biostatistics | University of California, San Francisco; The Social Innovation and Urban Opportunity Lab, Streetwyze | UCSF & San Francisco State University | Oakland, CA

## Abstract

**Background:**

Innovative data integration may serve to inform rapid, local responses to community needs. We conducted a mixed methods pilot study among communities of color or low-income in the San Francisco Bay Area amid the COVID-19 pandemic to assess a hypothesized data model to inform rapid response efforts.

**Methods:**

Between 2020–2021, we collected (1) qualitative data through neighborhood reports submitted via Streetwyze, a mobile neighborhood mapping platform; (2) survey data on social and economic circumstances; and (3) geospatial data among residents of three counties. Qualitative data were coded and then integrated with survey and geospatial data. We used descriptive analyses to examine participants’ experiences with food in their neighborhoods.

**Results:**

Seventy percent of participants reported food insecurity before and after the pandemic began in March 2020. Within neighborhood reports, *food* was the most frequently occurring sub-theme within the *Goods* and *Resources* parent themes (68% and 49% of reports, respectively). *Security* (88%), *resource programs* (88%), *outdoor space* (84%), and *equity* (83%) were more likely to be mentioned by participants who were food insecure compared to those who were not (12%, 12%, 16%, 17%, respectively). Mentions of food in neighborhood reports more often occurred in census tracts with lower socioeconomic status and more area-level food insecurity.

**Conclusion:**

Individuals who were food insecure reported a constellation of needs beyond food, including needs related to safety and greater social equity. Our data model illustrates the potential for rapid assessment of community residents’ experiences to provide enhanced understanding of community-level needs and effective support in the face of changing circumstances.

## INTRODUCTION

We recently described design aspects of a mixed methods, community-based participatory research (MMCBPR) pilot study within the California counties of Alameda, Contra Costa, and San Francisco during the COVID-19 pandemic that offered a unique approach to assessing community need.^1^ It incorporated continued feedback on participants’ experiences within their neighborhoods through Streetwyze, a mobile mapping platform designed with and for communities of color or low-income.^1^ During our study, we used emerging themes from these neighborhood reports to inform the development of repeated epidemiological surveys that assessed several domains of structural and social determinants of health (employment, healthcare access, housing, child/eldercare, transportation, food, finances, everyday discrimination) and well-being (COVID-19 stressors and physical/mental health). Recently, we also published an inductive analysis of neighborhood reports that describes three themes participants found particularly salient during the pandemic; innovation to foster community cohesion and establish informal networks; the value and importance of racial, ethnic, and culturally tailored services; and dignity in service.^2^ In addition to neighborhood reports (qualitative data) and surveys (quantitative data), we collected multiple forms of geospatial data including participants’ residence, locations associated with neighborhood reports, locations of resources listed in public resource directories and secondary neighborhood data. To further leverage the three data types collected, we now describe a data model that integrates them and illustrates the potential utility of doing so in the context of food access among study participants. The purpose of illustrating this data model is to present a real-world application of how sustained community engagement via a mobile platform and data integration can be used to identify localized, targeted, and specific areas of intervention to address salient community needs and expand community-identified solutions to unmet needs.

## METHODS

### Participant recruitment

We recruited a convenience sample of individuals residing in San Francisco, Alameda, or Contra Costa counties. Community-based recruitment strategies developed and implemented by Streetwyze and Roots Community Health Center included social media (e.g., Instagram, Twitter), printed advertisement (e.g., fliers distributed in community groups, posters in community clinics), and outreach presentations during existing virtual support group meetings run by Roots. We also outreached to existing Streetwyze users. A total of 75 participants were consented into the study. Of these participants, 51 completed a baseline survey, and 19 provided neighborhood reports (18 of whom also completed the baseline survey). All data were collected between September 1, 2020 and December 31, 2021.

### Data Collection

Qualitative data were collected as participant-submitted neighborhood reports in Streetwyze, a mobile mapping and SMS platform that allows for real-time mobile data collection via neighborhood reports, ratings, and reviews reflecting participants’ lived experiences.^1,2^ Within the Streetwyze platform, participants were directed to describe their experiences with resources and services during the pandemic through tailored focus questions (e.g., “Where is the safest and most affordable place for you to go to get the things you need?”, “Are there community-based organizations or neighbors who are making a difference in your life that you’d like to highlight?”). While participants were provided multiple format options to share their experiences (i.e., video, audio, or text), all reports we received in this study were text-based. A total of 236 neighborhood reports from 19 participants (range: 10 to 27 reports per participant) were received. Codebook development as well as formal inductive analysis of these reports have been published elsewhere.^1,2^ Briefly, the codebook was developed by a team of academic and community researchers to capture themes across neighborhood reports through preliminary assessment and discussion. A hierarchy of themes was developed to summarize six topical categories, which included: goods, resources, access, infrastructure, well-being, and infection control. Each of these “parent themes” included several sub-codes to reflect specific topics that were mentioned in the reports. For example, the ‘Access’ parent theme was subdivided into availability, convenience, price, orderliness, quality, security, and service (Supplemental Fig. 1). Each neighborhood report was independently coded by one community and one academic researcher; code conflicts were resolved by consensus among the full coding team.

Quantitative data were collected through an epidemiological survey (N = 51) via REDCap^3,4^ between January 1, 2021-October 31, 2021 and assessed participants’ circumstances prior to, and following, the COVID-19 pandemic shelter-in-place orders in the San Francisco Bay Area (March 2020). Survey domains included demographic characteristics (age, gender, race/ethnicity) along with social and structural determinants of health (employment, healthcare, housing, childcare, transportation, food, well-being, and financial circumstance). Surveys were drafted with questions and instruments sourced from the PhenX Toolkit and other published sources;^5–22^ the full baseline and follow-up surveys are available as Supplemental Methods and include source references relevant to specific survey items. In some cases, instruments were added (e.g., COVID-related safety, coping) or adapted (e.g., pandemic-related concerns) to our study based on integration of results of qualitative data analysis according to a convergent mixed methods approach.^1^ Food insecurity was assessed by adapting the six-item standard measure from the U.S. Department of Agriculture Economic Research Service (Supplemental Methods).^20^

Geospatial data. All (100%) participants who completed the baseline survey provided a full residential address (n = 50) or cross-streets of residence (n = 1). Addresses and cross-streets were geocoded to latitude/longitude coordinates using ArcGIS; census tract identifiers for 2010 census geographies were then appended. Measures of census tract-level neighborhood SES (nSES) and percent food insecure were obtained from UCSF Health Atlas.^23^ Neighborhood SES was previously created with principal components analysis of measures related to census tract-level income, occupation, education, and housing with data from the American Community Survey (2013–2017); quintiles are based on the distribution of index scores among all census tracts in the state of California.^24^ The proportion of individuals within a census tract experiencing food insecurity was from Feeding America’s Map the Meal Gap study (2016 and 2017).^25^

Neighborhood reports provided via the Streetwyze mapping platform are geocoded to latitude/longitude coordinates within the Streetwyze application and were then assigned to 2010 census tracts.Public resources described via Alameda, Contra Costa, and San Franciscocounty directories were obtained from in March 2021; the address of each resource was geocoded using Google API; census tract identifiers were then appended. The frequency of resources per capita was calculated for each census tract using population estimates from Census 2010.^26^

### Data Analysis and Integration

Frequency of code applications across each parent theme were used to summarize qualitative data using descriptive statistics across all domains of structural and social determinants of health for the total sample, as well as by self-reported food insecurity.

**Figure 1:**
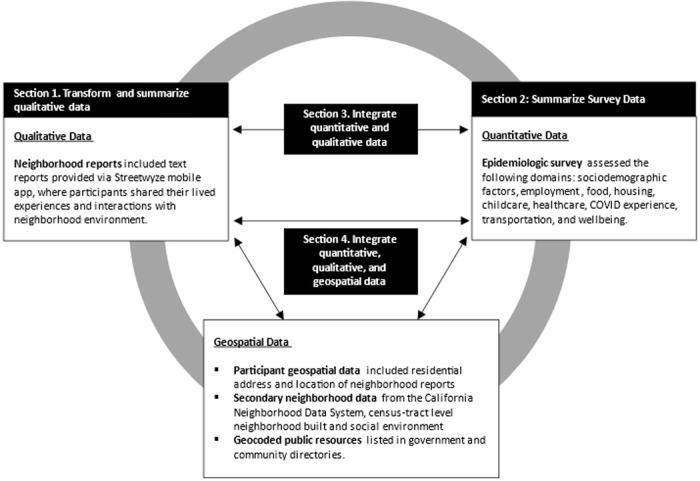
Data types and descriptions, data analysis, and data integration

We explored several data integration approaches illustrated by the data model in Fig. 1. First, we transformed qualitative reports into quantitative data at the participant level by creating an indicator for whether each code was mentioned more than average. Frequencies of these transformed qualitative data were then summarized by food insecurity status as self-reported by participants via survey. We found that the compound effects of experiencing multiple forms of stressors can influence an individual’s wellbeing. Therefore, we additionally evaluated the distribution of frequently mentioned codes within parent themes which appeared to differ substantially between those who were never and ever food insecure (i.e., resources, wellbeing, and infrastructure). Finally, we integrated geospatial measures by (1) overlaying the locations where participants reported receiving food resources via survey atop a base map illustrating the proportion of food resources for each census tract and (2) illustrating locations where participants mentioned food resources in neighborhood reports atop census tract-level base maps illustrating nSES and proportion of residents who experienced food insecurity. All maps were created with ArcGIS using shapefiles for Census 2010 tract boundaries available from the National Historical Geographic Information System.^27,28^

## RESULTS

Data types and our data integration approach are illustrated in Fig. 1 (Subsections below correspond to number boxes in Fig. 1).

### 1. Food experiences in neighborhoods: Summary of transformed qualitative data

Of the 236 qualitative reports collected from 19 participants, the most frequently mentioned parent themes were *access* (61%) and *infection control* (59%) while the least common were *infrastructure* (11%) and *well-being* (5%). Frequencies of sub-themes within each of the parent themes is presented in Supplemental Fig. 2. *Food* was the most frequently mentioned sub-theme within the parent themes of *resources* (49%) and *goods* (68%).

### 2. Participants circumstances around food access: Summary of survey data

A total of 51 participants completed the baseline survey (Table 1). Most participants lived in Alameda County (85.7%), were between 18–45 years of age (77.6%), were women (74.0%), and had less than 6 months of household savings (68.6%). Most participants reported changes in employment (68.6%) and transportation (60.8%) from before to after the pandemic began in March 2020. On average participants reported 7.7 stressors related to the COVID-19 pandemic, and 202.5 experiences of discrimination in the past year. Over 70% of participants reported food insecurity in both pre-and post-pandemic time periods.

In the period after the pandemic began, 65% of participants reported receiving reduced price or free food resources. The most common resources used were from non-profit organizations (43%, including Meals on Wheels and food banks), government food assistance programs (22%, including CalFresh, the Special Supplemental Nutrition Program for Women, Infants, and Children, or the pandemic Electronic Benefit Transfer (P-EBT) program), and free meals through schools (31%).

### 3. Intersections of individual experiences and neighborhood food experiences: Integrating neighborhood reports and survey responses

**Figure 2:**
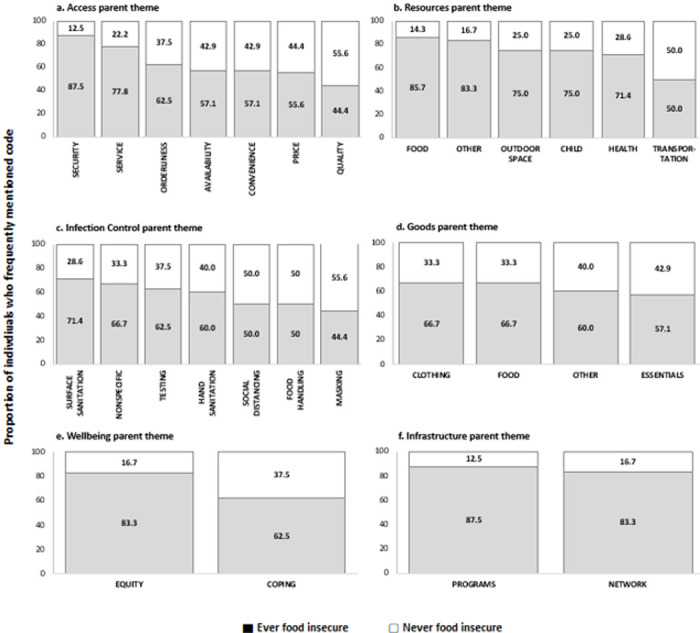
Distribution of frequently mentioned codes within each parent theme based on whether individuals reported food insecurity via survey responses

An illustrative integration of qualitative and quantitative data is displayed in Fig. 2. For each of the parent themes, the distributions of frequently mentioned codes in qualitative reports are presented according to self-reported food insecurity (ever vs. never food insecure). Most mentions of social and resource programs (87.5%) and informal networks (83.3%) were among participants who were food insecure (12.5% of mentions of resource programs and 16.6% of mentions of informal networks were among those who were not food insecure). Mentions of programs and networks were not specific to food; they include mentions of healthcare, childcare, and community-based organizations (e.g., organizations providing legal assistance). Within the Access parent category, mentions of sub-themes among those who were food insecure ranged from 87.5% of the total mentions of quality of goods to less than 45% of the total mentions of security or safety (compared to 12.5% of the total mentions of quality of goods and 55% of the total mentions of security among those who were not food insecure). These differences may indicate the priority of concerns among participants who are food insecure.

**Figure 3:**
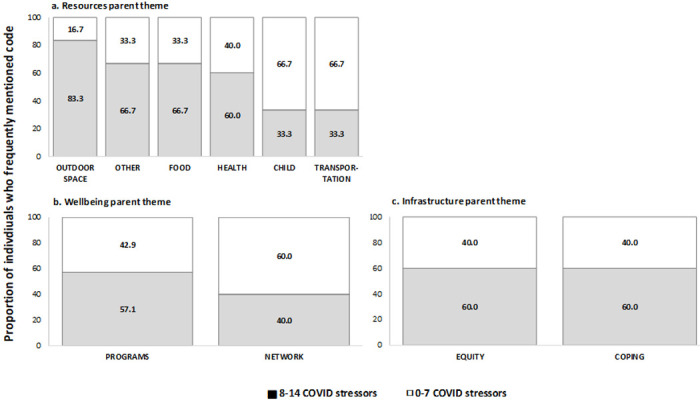
Distribution of frequently mentioned codes within (a) resources, (b) infrastructure and (c) well-being by reported number of COVID-19 stressors, among those who ever reported food insecurity

**Figure 4:**
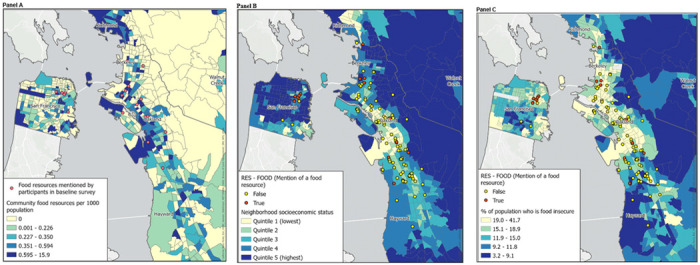
Geospatial integration of participant qualitative and quantitative data

Figure 3 presents the frequency of themes mentioned *among* participants who were ever food insecure according to the number of pandemic-related stressors reported, categorized as below (< 8) and above (>/=8) the average number of stressors among the total participant population. Those who reported food insecurity and >/= 8 pandemic-related stressors had a higher proportion of reports mentioning outdoor space (83.3%), food (66.7%), and health (60.0%) resources; programs and solutions (57.1%); and experiences related to equity (60.0%) and coping (60.0%) compared to those who reported fewer than 8 stressors (Fig. 4). Participants who were food insecure and had >/= 8 pandemic-related stressors, on the other hand, were less likely to mention child or transport resources.

### 4. Locating food experiences: Integrating neighborhood reports, survey responses, and geospatial measures

Figure 4 presents strategies for integrating geospatial data with other data types within the domain of food security and food access. In Fig. 4a, neighborhood food resources reported by participants via survey are located over the density of food resources as reported by public resource directories. The food resources that participants reported utilizing appeared to be located within census tracts with a higher density of food resources. In Fig. 4b and 4c the location of qualitative reports mentioning food resources are overlayed on tract-level neighborhood socioeconomic status (4b) and the proportion reporting food insecurity (4c). In general, it appears that neighborhood reports mentioning food resources occurred in census tracts with lower nSES and higher proportions of food insecurity.

## DISCUSSION

We used data collected through this MMCBPR pilot study to illustrate how several data elements can be integrated to describe participants’ experiences around social determinants of health using the domain of food insecurity as an example. By integrating qualitative data from written neighborhood reports, surveys, and geospatial data, we present a data model with the potential to identify specific, targeted needs and resources that emerge from participants’ experiences. In our example, data integration indicates how participants who have ever experienced food insecurity (before or after the beginning of the pandemic) were more likely to also report employment, housing, and healthcare changes during the pandemic and more likely to report everyday discrimination. Similarly, those reporting food insecurity were much more likely to mention resources and programs of any kind and to report experiences related to equity in their neighborhoods.

While this report is focused on food insecurity, it is intended to serve as an illustrative example of how this data model can inform tangible solutions across domains of structural and social determinants of health. For example, we found that those reporting both food insecurity and a higher number of other pandemic-related stressors had higher mentions of resources, programs, potential solutions, and issues related to equity and coping. This suggests that more comprehensive wrap-around services are needed for individuals who already experience stressors related to food insecurity. In addition, participants who mentioned food in neighborhood reports and who had many COVID-related stressors suggested areas for resource development related to outdoor space and health. Thus, a multilevel response to this need might include 1-community food service organizations serving as a point of contact for individuals who may benefit from other resources (healthcare and public health information) and 2-investment in outdoor spaces as sites to promote physical and mental health as well as deliver tangible health resources. Similarly, this observation stresses the importance of cross-institutional communication and connectivity to alleviate the administrative burden experienced by those who access multiple social services.

In our study, results from maps largely serve to validate the congruence of information across our data sources (e.g., mentions of food occurring most frequently in neighborhoods with higher area-level food insecurity). While we are reassured in the capacity for transformation of neighborhood reports to align with other measures of food access or experience, integration of reports or survey data with geospatial data would be most powerful when it contributes to new or emerging understanding of communities’ needs. Doing so may require smaller geographic units of analysis with larger numbers of community residents contributing reports and survey responses. For example, emergence of themes related to (non-COVID) safety in the vicinity of an existing food resource may be an early signal of an emerging access barrier or relatively frequent positive mentions of racial equity may identify exemplary aspects of existing programs. And of course, new mentions of needed resources may indicate specific areas with emerging need. We will continue to develop the relevancy of our data model to hyper-local assessment of barriers and solutions in ongoing and future studies.

A limitation inherent to our data model is that integration across data sources inevitably reduces cell sizes. Specifically, because participants were provided with multiple options to share their experiences and perspectives, there was limited overlap between data sources. This could lead to an overreliance on making inferences based on single data sources rather than on integration. Despite that, using a data integration model can help overcome the limitations of small sample sizes by leveraging information across sources of data to reinforce the validity of each. For instance, the fact that there were more mentions of food resources in qualitative neighborhood reports among those reporting food insecurity in the epidemiologic survey bolsters the validity of both data sources. Due to the small size of the participant population in this pilot study, we were unable to examine results according to individual-level factors such as race, ethnicity, immigration status, LGBTQA + status, and other individual social factors that impact individuals’ vulnerability to structural oppression. Targeted data collection to specific populations where appropriate or increasing participation would allow us to better represent those with intersecting identities.

Despite these limitations, there are several key benefits of this approach to data collection compared with other approaches that have been employed. First, collecting qualitative data using a mobile platform combined with brief, focused survey questions simultaneously with geographic information allows for continued participation compared with longer forms of data collection (e.g., in-depth interviews). Additionally, because the Streetwyze platform allows participants to share their feedback and experiences in real-time and within the context of their own neighborhood experiences, the barrier to participation is lower. This allows for sustained and low-effort participation and can make research more accessible for more diverse people and experiences.

Overall, we have demonstrated the feasibility of simultaneous, multi-modal data collection, which can be scaled to allow for a wider scope of inquiry, along with a tangible model for data integration with the potential to identify salient needs emerging from communities. The combined analysis of quantitative, qualitative, and geospatial data can be adapted by community organizations, policy makers, and researchers to improve the implementation of policies and services over time by periodically tracking and analyzing residential reports and sustaining community participation. This effort is in line with our broader intent to facilitate community-led research and community ownership of data that generates local knowledge, investment, and progress (Akom, Hope Hassberg, and Cruz; pending book chapter).

## Figures and Tables

**Table 1:** Frequency distribution of sociodemographic characteristics of study participants according to self-reported food insecurity (Survey data)

	Total participants N = 51	Food insecure ever N = 40	Persistent food insecurity N = 34
**% or mean (standard deviation)**			
**Total**		74.5	70.6
**County**			
Alameda	85.7	86.8	87.5
San Francisco/Contra Costa	14.3	13.2	12.5
**Age group (in years)** ^1^			
18–45	77.6	81.6	84.4
46+	22.5	18.4	15.6
**Race or ethnicity** ^1,2^			
Asian American	24.0	23.1	24.2
Black or African American	28.0	25.6	30.3
Hispanic	28.0	33.3	30.3
White and Other	20.0	18.0	15.2
**Gender** ^1^			
Man	24.0	23.1	24.2
Woman	74.0	74.4	72.7
**Household savings**			
Less than a month	35.3	42.5	47.1
1–2 months	33.3	37.5	35.3
3–6 months	17.7	12.5	11.8
More than 6 months	13.7	7.5	5.9
**Employment change** ^3^	68.6	75.0	79.4
**Housing change** ^3^	49.0	55.0	55.9
**Transportation change** ^3^	60.8	60.0	61.8
**Healthcare change** ^3^	29.4	35.0	35.3
**Number of COVID-19 stressors** ^4^	7.7 (4.1)	8.2 (4.1)	8.2 (4.1)
**Good/very good/excellent physical health** ^5^	78.4	80.0	79.4
**Good/very good/excellent mental health** ^5^	58.8	57.5	52.9
**Number of past year discriminatory experiences**	202.5 (376.9)	240.8 (414.4)	274.6 (441.2)

1. Missing data: age (n = 1), race or ethnicity (n = 1), gender (n = 2)

2. The “other” category of race or ethnicity includes Middle Eastern/North African and American Indian/Alaska Native. Financial circumstance was evaluated by asking participants how long they could maintain their lifestyle with their current savings.

3. Changes to employment, housing, transportation, and healthcare were assessed by asking participants if these factors change following pandemic restrictions in March 2020.

4. Participants selected from a list of 17 different possible COVID-19 stressors, including concerns for health of self, health of family members, financial concerns, impact on work, impact on child(ren), impact on community, impact on relationship with adult family members, access to food, access to baby supplies, access to personal care products or household supplies, access to healthcare, access to housing, ability to parent how I want, ability to care for older adults or people with disabilities, social distancing or being quarantined, transportation and safety, or something else.^21^

5. PROMIS Global Physical Health and Global Mental Health^5,6^

6. The number of past year discriminatory experiences was calculated based on the Everyday Discrimination Scale, where the sum total responses were weighted for each participant to capture the annual chronicity of discrimination experiences.^22^

## Data Availability

The datasets generated and/or analyzed during the current study are not publicly available due to community preference but are available from the corresponding authors on reasonable request.
